# Cross-Linked α-Synuclein as Inhibitor of Amyloid Formation

**DOI:** 10.3390/ijms241713403

**Published:** 2023-08-29

**Authors:** Nikoletta Murvai, Gabriella Gellen, András Micsonai, Gitta Schlosser, József Kardos

**Affiliations:** 1 Department of Biochemistry, Institute of Biology, ELTE Eötvös Loránd University, H-1117 Budapest, Hungary; 2 ELTE—Functional Nucleic Acid Motifs Research Group, Department of Biochemistry, Institute of Biology, ELTE Eötvös Loránd University, H-1117 Budapest, Hungary; 3 MTA-ELTE Lendület Ion Mobility Mass Spectrometry Research Group, Department of Analytical Chemistry, Institute of Chemistry, ELTE Eötvös Loránd University, H-1117 Budapest, Hungary; 4 Doctoral School of Molecular Cell and Immune Biology, University of Debrecen, Egyetem tér 1, H-4032 Debrecen, Hungary; 5 Department of Biophysics and Cell Biology, Faculty of Medicine, University of Debrecen, Egyetem tér 1, H-4032 Debrecen, Hungary; 6 ELTE NAP Neuroimmunology Research Group, Department of Biochemistry, Institute of Biology, ELTE Eötvös Loránd University, H-1117 Budapest, Hungary

**Keywords:** amyloidogenic proteins, intrinsically disordered proteins, cross-linking mass spectrometry, MD simulations, inhibition of amyloid formation

## Abstract

The aggregation and amyloid formation of α-synuclein is associated with Parkinson’s disease and other synucleinopathies. In its native, monomeric form α-synuclein is an intrinsically disordered protein represented by highly dynamic conformational ensembles. Inhibition of α-synuclein aggregation using small molecules, peptides, or proteins has been at the center of interest in recent years. Our aim was to explore the effects of cross-linking on the structure and aggregation/amyloid formation properties of α-synuclein. Comparative analysis of available high-resolution amyloid structures and representative structural models and MD trajectory of monomeric α-synuclein revealed that potential cross-links in the monomeric protein are mostly incompatible with the amyloid forms and thus might inhibit fibrillation. Monomeric α-synuclein has been intramolecularly chemically cross-linked under various conditions using different cross-linkers. We determined the location of cross-links and their frequency using mass spectrometry and found that most of them cannot be realized in the amyloid structures. The inhibitory potential of cross-linked proteins has been experimentally investigated using various methods, including thioflavin-T fluorescence and transmission electron microscopy. We found that conformational constraints applied by cross-linking fully blocked α-synuclein amyloid formation. Moreover, DTSSP-cross-linked molecules exhibited an inhibitory effect on the aggregation of unmodified α-synuclein as well.

## 1. Introduction

Cross-linking, the formation of covalent bonds between protein molecules or parts, plays a crucial role in shaping protein structure and modulating their function. In the case of amyloidogenic proteins, which are associated with various neurodegenerative disorders, cross-linking and alterations in conformation can significantly influence the propensity for protein aggregation [1,2,3,4,5].

Cross-linking occurs when reactive chemical groups, such as cysteine or lysine residues, form covalent linkages between protein regions or molecules. These inter- or intramolecular bonds restrict the conformational flexibility of proteins, resulting in a variety of structural consequences. Cross-linked proteins often adopt more compact and rigid structures, altering their overall shape and accessibility to other molecules. Consequently, the functional properties of proteins, including enzymatic activity, binding affinity, and cellular interactions, can be profoundly affected by cross-linking [6,7,8,9].

Amyloidogenic proteins are a class of proteins that can misfold and aggregate into insoluble amyloid fibrils, a hallmark of many neurodegenerative diseases, such as Alzheimer’s and Parkinson’s disease. The aggregation process is highly dependent on the conformational states of these proteins. Cross-linking of amyloidogenic proteins can occur through various mechanisms, including oxidative stress, post-translational modifications, or through the action of molecular chaperones [1,10,11,12,13,14]. The presence of cross-links within amyloidogenic proteins can influence the rate, extent, and stability of protein aggregation. Cross-linking often promotes the formation of larger protein aggregates, altering the kinetics of fibril formation. These cross-links can stabilize intermediate oligomeric species, which are believed to be the toxic entities responsible for cellular dysfunction and neuronal damage in neurodegenerative diseases. Additionally, cross-linking may hinder the clearance of aggregated proteins by the cellular proteostasis machinery, further exacerbating the pathological consequences [3,15]. Cross-linking could also be a potential approach for inhibition of aggregation processes. By creating spatial restraints within the protein’s folding landscape, cross-linking aims to restrict the conformational flexibility necessary for aggregation. The introduction of appropriate steric constraints could limit the formation of the β-sheet-rich conformations typically associated with aggregation-prone states. By specifically interacting with the regions prone to aggregation, the cross-linking agents prevent the formation of intermolecular contacts required for fibril assembly, effectively inhibiting the protein’s pathogenic behavior. The steric constraints imposed by intramolecular cross-linking not only can disrupt the aggregation-prone conformations but also confer enhanced stability to the protein, thereby reducing its susceptibility to proteolytic degradation. This added stability can further contribute to the inhibition of aggregation processes and provide potential therapeutic benefits [3,16].

In recent years, the cross-linking mass spectrometry (XL–MS) technique has emerged as a widely applied method for studying the structure of proteins that are difficult to investigate with conventional methods (e.g., NMR spectroscopy and X-ray crystallography), such as intrinsically disordered proteins or proteins with disordered regions. Cross-linking agents usually contain two reactive groups that are connected by a spacer with specific length. The spacer provides a distance information between amino acid residues of a protein or protein–protein partners which are hereby covalently cross-linked together. The cross-linked amino acids in the protein chains are then analyzed generally by bottom-up proteomic approaches using LC/ESI–MS/MS (liquid chromatography/electrospray ionization–tandem mass spectrometry).

The most commonly used cross-linkers are amine-reactive *N*-Hydroxysuccinimide (NHS) esters, which target primarily lysine side chains. Many different NHS ester-based cross-linkers with various solubilities, spacer arm lengths, and gas-phase cleavability are available commercially. The most frequently used NHS cross-linker is the water-soluble, non-gas-phase cleavable BS3 (bis(sulfosuccinimidyl)suberate) [17] with a sulfonic acid group at both reactive ends and a spacer arm length of 11.4 Å. The sulfonic group is incorporated for increased water solubility. Additionally, cross-linking agents with longer (e.g., BS(PEG)5 (PEGylated bis(sulfosuccinimidyl)suberate) of 21.7 Å) or shorter (e.g., EDC (1-ethyl-3-(3-dimethylaminopropyl)carbodiimide hydrochloride) with zero length) distance constraints can foster the reconstruction of a protein 3D structure. Furthermore, targeting not only lysine residues, but for example the more acidic aspartic and glutamic acids like in the case of the EDC cross-linker, can provide complementary distance information to the lysine reactive cross-linkers [18]. Other popular cross-linkers are DSP [19] (dithiobis(succinimidylpropionate)) and its water-soluble sulfo-NHS ester variant, the DTSSP, (3,3′-dithiobis(sulfosuccinimidyl propionate)) with a spacer arm length of 12.0 Å, which are cleavable under reducing conditions in solution or in gas-phase during collision induced dissociation (CID). Gas-phase cleavability of a cross-linker may facilitate the unambiguous identification of the cross-linked peptide pairs through the production of characteristic fragmentation patterns in the MS2 spectra [20].

α-Synuclein (α-syn) is considered an intrinsically disordered protein (IDP) and its monomeric form is characterized by highly dynamic conformational ensembles. Aggregation and amyloid formation of α-syn is associated with Parkinson’s disease and other synucleinopathies [21,22]. In this work, we try to map the monomeric, natively disordered structure of α-syn by cross-linking and investigate the effect of steric constraints introduced by cross-linking on the aggregation and fibrillation of the protein. First, we carried out in silico structural analysis to predict the potential sites for cross-linking and their potential inhibitory effect on fibrillation. Then, we cross-linked monomeric α-syn intramolecularly with several cross-linkers and determined the location of the cross-links by mass spectrometry. Cross-linked α-syn samples were tested for their aggregation properties and for their inhibitory effect on the fibrillation of unmodified α-syn.

## 2. Results

### 2.1. Analysis of α-Synuclein Structures for Potential Cross-Linking Residue Pairs

**Figure 1:**
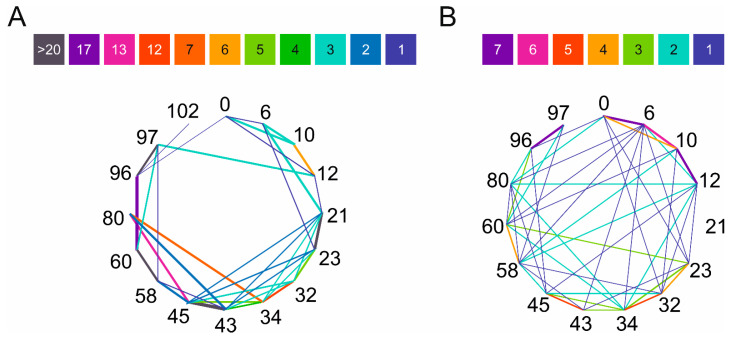
Network diagram of Lys pairs within 15 Å distance in different α-syn forms. (**A**) Lysines in the amyloid structures were surveyed on the deposited 64 PDB structures (Supplementary Table S1). (**B**) Lysines that fall within 15 Å and thus might be subjected to cross-linking in the eight representative model structures of the monomeric state of Chen et al. [24] (see also Supplementary Figure S1). Color coding shows the frequency of the Lys-Lys pairs in the structures in the 64 amyloid or 8 monomeric structures. The results reveal the structural diversity of α-syn monomers and show large differences in the pattern between the amyloid and monomeric states. The N-terminal amino group is also included into the analysis and is indicated with ‘0’.

High resolution structures of amyloid forms of α-synuclein have been determined by solid-state NMR and cryo-EM and are available in PDB. To examine the amyloid forms, we collected 64 accessible, deposited, α-synuclein fibril structures from PDB (see Supplementary Table S1 for PDB entries). However, structural characterization of the natively unfolded monomeric protein is more difficult. Cross-links with a triazine (TATA) were identified using LC–MS/MS and used to model the hypothesized molten globule state of α-syn [23]. Chen et al. studied the conformational ensemble of monomeric α-synuclein using FRET experiments and MD simulations and distinguished eight structures that represent well the overall conformational ensemble [24]. Using the amyloid structures and the structures representative of the native state, we searched for potential side chain pairs for cross-linking and checked if they exclusively appear in the monomeric structures and are incompatible with the amyloid forms. Considering the length of the linkers of the chosen cross-linkers, we have set the upper limit as 15 Å for residue pairs. The most commonly used cross-linkers are amine-reactive ones, which target primarily lysine side chains. Thus, we have taken into consideration the Lys side chains’ distance and, since the N-terminal is also reactive during the cross-linking reactions, the analyses have also contained this free amine. Results on the amyloid structures and on the eight monomer structures are presented in Figure 1 and in Supplementary Figure S1. Our goal was to see whether characteristic correlation can be observed for the distinct structures based on the obtained positions. In the case of the studied fibril structures, the most common proximities are 21–23, 43–45, 58–60, 96–97, 60–96, 45–80, 32–34, and 34–80, while in the eight representative monomer forms these are 0–6, 10–12, 96–97, 6–10, 32–34, and 43–45. We have to note the higher diversity and number of cross linkable Lys-Lys pairs in the monomeric structures. Furthermore, large number of the Lys-Lys pairs of the monomeric structures cannot be found in the amyloid forms. This observation supports our idea that proper cross-links of the monomeric α-syn might be incompatible with the amyloid structures and thus may protect the molecule against aggregation.

### 2.2. Chemical Cross-Linking and Identification by Mass Spectrometry

Cross-linkers used in our study were BS3, BS(PEG)_5_, DTSSP, and EDC (Supplementary Figure S2) with amine reactivity with the exception of EDC. α-Syn contains 15 Lys residues and the N-terminal amino group resulting in 120 possible single cross-link combinations within the molecule. The cross-linking reactions’ conditions were optimized for the formation of intramolecular connections only, which have been confirmed by SDS PAGE (Supplementary Figure S3). The cross-linking reactions were also examined using HPLC and compared to unmodified α-syn control. The results reveal the high efficiency of the cross-linkings with a low amount of remaining unmodified protein. The broad bands of cross-linked samples suggest a large heterogeneity in cross-linking (Supplementary Figure S4).

**Figure 2:**
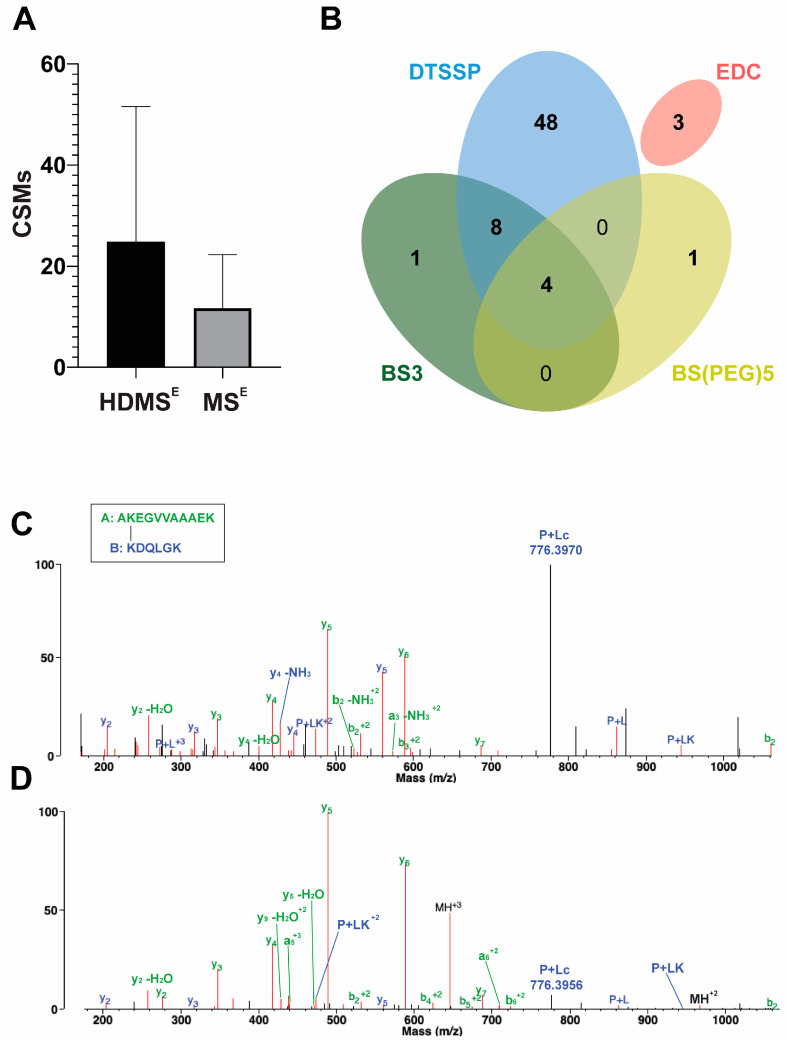
Cross-link identifications using HDMS^E^ and MS^E^ approaches. (**A**) Comparison of the mean of identified cross-link spectra matches (CSMs) in different biological replicates of DTSSP-cross-linked samples between HDMS^E^ and MS^E^ approaches. In the case of HDMS^E^, when one cycle of ion mobility separation was applied, about twice more CSMs were identified as compared to the MS^E^ method with no ion mobility separation. (**B**) Overlap of identified unique cross-links using cross-linkers with different spacer arm lengths and amino acid reactivities by both HDMS^E^ and MS^E^ approaches. By applying these various types of cross-linking reagents, it became possible to obtain complementary distance information between different amino acid residues, which could enhance 3D protein structure predictions for monomeric α-syn conformations. (**C**,**D**) Comparison of the MS/MS spectra of the cross-link between K12 and K97 lysines identified after (**C**) HDMS^E^ and (**D**) MS^E^ approaches visualized in Protein Prospector’s display mode. In HDMS^E^ mode, it was possible to determine more fragments of both peptides of the cross-link in comparison to the MS^E^ mode. Furthermore, an intensive peak appeared at 776.3970 *m*/*z*, which refers to the Peptide B with a half linker which was formed upon the dissociation of the S–S bond. The score for this cross-link was 43.0. In MS^E^ mode, fewer fragments were identified from Peptide B, and the score of this identification was 31.0. The fragment of the S–S bond cleavage is less pronounced in this case. Peptide A was K(+DSP)DQLG (11–21) and Peptide B was AK(+DSP)EGVVAAAEK (97–102).

The introduced cross-links’ positions were identified by LC–MS/MS with and without cyclic ion mobility separation (IMS) before fragmentation and mass analysis. By applying a single pass ion separation cycle in the novel cyclic IMS system, on average, twice as many DTSSP cross-link spectra matches (CSMs) were identified using Protein Prospector v.6.4.9 software than without IMS (Figure 2A). In parallel, identification of the linear peptides also slightly increased, but the overall protein sequence coverage remained the same in both MS techniques. The average overlap of unique cross-link identifications between HDMS^E^ and MS^E^ methods per biological replicates of the DTSSP-cross-linked samples was ~48%. HDMS^E^ acquisition mode generated more fragments of both peptides of the cross-linked peptide pair; furthermore, the cleavage of the S–S bond of the DTSSP cross-linker upon CID fragmentation (P + Lc ion) was also more defined (Figure 2C). An increased number of fragments from the linker further improves unambiguous identification of cross-linked peptides and increases the score calculated by the algorithm. In contrast, during MS^E^ acquisition mode less fragmentation of the S-S bond occurred, and a lower number of other linker-containing fragments were observed and cleaved at the peptide bond originating from the modified lysine from the opposing peptide (PL ion) and its tetrahydropyridine modification (PLK ion) (Figure 2D). Furthermore, among the 120 possible combinations of amine cross-links, we could determine 62 unique linkages with NHS ester cross-linkers of different lengths. This number was increased by three additional identifications using the zero length EDC cross-linker, which creates links between lysine residues and aspartic acid or glutamic acid (Figure 2B).

Using the unmodified α-syn control sample, we checked if trypsin digestion and the following LC–MS/MS procedure results in α-syn fragment identification that covers with equal frequency the entire sequence. This is needed to confirm that cross-linking frequencies identified by MS can be used as quantitative measure. We observed a smooth coverage of the sequence, except the for the 81–96 fragment that appeared with double density (Supplementary Figure S5). Because cross-linking in the 81–96 region just occurred at the lowest frequencies, the observed higher sensitivity of the MS technique for this region will not change the tendencies. Thus, we concluded that we could use the cross-linking frequencies observed by MS as quantitative measures.

In our experiments with α-syn, the DTSSP cross-linker has proven to be the most reactive and gave most of the cross-link identifications. Therefore, we chose this linker for further experiments and mapping of the monomeric structure of α-syn. To characterize the efficiency of the cross-linking reaction by DTSSP, we measured the unmodified and the cross-linked samples without digestion and fragmentation by LC–ESI–MS (Supplementary Figures S6 and S7). The results revealed that a large portion of the molecules had single cross-linking with or without an extra monolink (>50%). In a small fraction, unmodified (~10%) and several cross-linked molecules were also observable.

### 2.3. Comparison of Experimental Cross-Linking Results with Distance Analysis on Amyloid Structures

**Figure 3:**
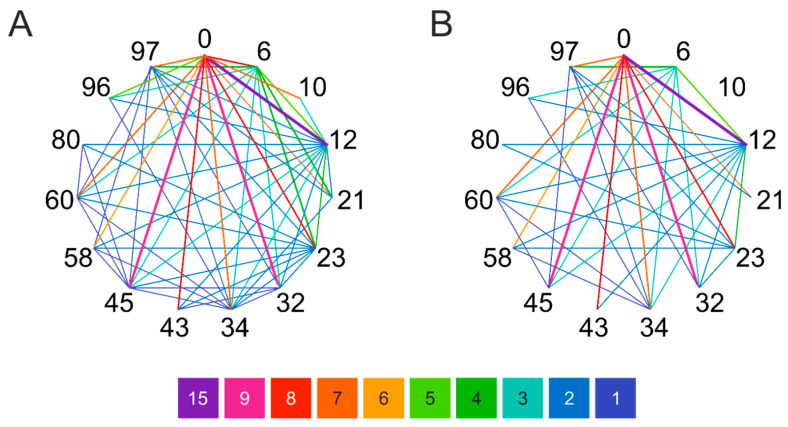
Lys pairs of α-syn cross-linked by DSSP. (**A**) Detected DTSSP cross-links and frequencies of their occurrence (colored, purple to blue) in the measured α-syn samples. (**B**) Cross-links out of those in (**A**) that cannot be realized in the amyloid structures. The proportion of these cross-links is around 90% of all. The N-terminal amino group is also included into the analysis and is indicated with ‘0’.

Considering the information gained using MS measurements about the introduced cross-links within the α-syn molecule, DTSSP has shown to be the most promising cross-linker with many created connections and the most pronounced effect (see Section 2.5). According to experimental data, the N-terminal was prone to react with ε-nitrogen of Lys residues via DTSSP. However, many other positions could react with different frequencies shown in Figure 3A. The positions most involved in the cross-linking reactions by DTSSP are 0–12, 0–32, 0–45, 0–6, 0–23, and 0–43 (N-terminal α-amino group is labelled as 0). Supplementary Table S2 shows the frequency of the various cross-links identified by MS. We assigned the 60 amine pairs cross-linked by DTSSP to the 64 α-syn amyloid structures deposited in PDB and sorted out those that are within cross-linking distance (15 Å) in the amyloid structures and thus are amyloid compatible. The frequencies of these cross-links were low. The remaining 37 Lys pairs are exclusive for the monomeric α-syn (Figure 3B) and considered as not amyloid compatible. Intriguingly, they represent ~90% of the cross-links in overall frequency. The most frequently formed cross-links belong to this group. We have to note that the 0–12 Lys pair can be found within cross-linking distance only in one of the 64 amyloid structures but cross-linked at high frequency in the monomer samples.

### 2.4. Distance Analysis of Amino Groups in MD Simulation of Monomeric α-Synuclein

**Figure 4:**
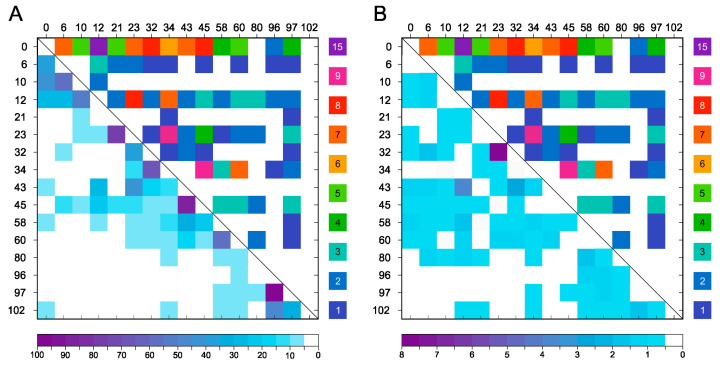
Frequency of amino group pairs of α-syn that are within the cross-linking distance. (**A**) Distances were measured on 5001 frames of 1 μs MD trajectory between all amino groups and the percentages of the time (frames) when they were within cross-linking distance are presented in the lower left half. Upper right part presents the experimentally identified DTSSP cross-link frequencies in number of identifications using mass spectrometry (Supplementary Table S2). (**B**) Amine pairs within cross-linking distance were filtered further for those that are sterically available for each other, i.e., the space between them is not occupied by other molecule parts. At the left lower part, frequencies of cross-linkable amine pairs are shown in % of frames in the overall trajectory. Upper right part experimentally identified DTSSP cross-link frequencies. The N-terminal amino group is also included into the analysis and is indicated with ‘0’.

Comparing the experimentally formed 60 DTSSP cross-links to the potential cross-links identified by the amine–amine distances in the representative structures of Chen et al. [24] (Figure 1B), we found that only 33 cross-links can be assigned to them. This important observation reveals that the eight monomer α-syn structures are insufficient to represent the entire conformational ensemble of the intrinsically disordered α-syn monomer. To provide a better in silico structural characterization, MD simulations of monomer α-syn were carried out using GROMACS software package (v.2020.3) with an AMBER-ff99SB*-ILDNP force field and a TIP4P water model that are suitable for IDPs [25]. A total of 5001 frames of the overall 1 μs trajectory were analyzed for amino group distances (see Section 4). We found that all the 120 possible amine pairs happened to be located within 15 Å distance in at least a few frames in the trajectory. The frequencies of potentially cross-linkable pairs with less than 15 Å distance is shown in Figure 4A. However, comparing these frequencies to that of the 60 experimentally identified cross-links (Supplementary Table S2), we found no correlation. To improve the identification of potential cross-links by MD, we sorted out the amine pairs located within 15 Å for the condition that they are sterically available for each other, i.e., the space between them is not occupied by other molecule parts (see Section 4). Still, most of the possible 120 cross-link pairs fulfilled this criterion. Although compared to the simple distance analysis (Figure 4B) their frequencies are changed, there is still no real correlation with the experimental results. This reveals that the disordered α-syn chain occupies a large conformational space and mostly all the amine pairs come to cross-linking distance with no steric hindrance for some part of the simulations. However, in the case of α-syn, the real, experimental cross-linking probabilities cannot be deduced from MD simulations applying such a simple investigation.

**Figure 5:**
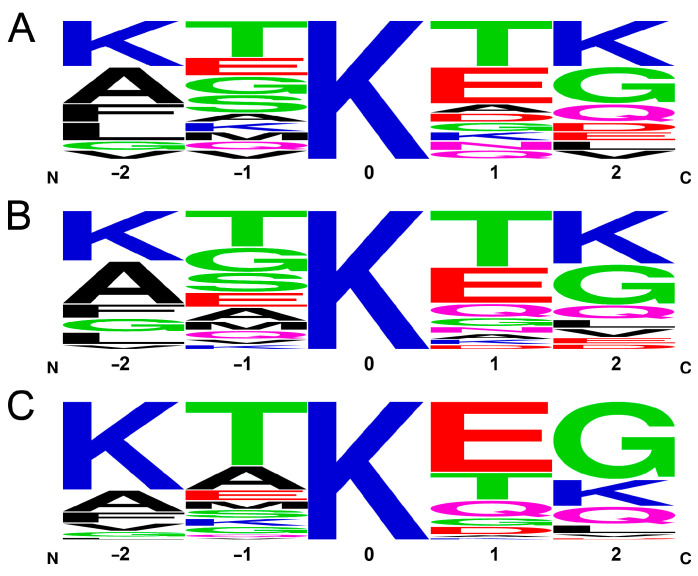
Sequential environment of lysines. (**A**) Frequency logo of the 15 pentapeptides of α-syn with Lys in the center. (**B**) Frequency logo weighted by occurrence within cross-linkable distance to another amine with no steric hindrance in the MD trajectory. (**C**) Frequency logo weighted by the occurrence in cross-links identified by MS.

A possible explanation for the inability of MD to predict the cross-linking probabilities is that the cross-linking efficiency is rather determined by the chemical reaction rate, which is dependent on the *pK*_a_ of the amino group because the unprotonated -NH_2_ form reacts with DTSSP. This hypothesis is supported by the fact that 23% of the cross-links (14 out of 60) identified by MS with an overall frequency of 41% is formed by the N-terminal amino group, which has a significantly lower *pK*_a_ value than those of the lysines (~9 vs. ~10.5) and thus is more reactive at the pH used in the cross-linking reaction. The *pK*_a_ value of a lysine ε-amino group will depend on its local environment, which is mainly given by the neighboring side chains in the amino acid sequence. Although we cannot calculate the *pK*_a_ of the amino groups here, we are able to examine how the primary sequence affects the cross-linking frequency. To address this question, we examined the frequency of the two–two neighboring residues before and after the lysines in the sequence. We chose two–two residues, because, depending on the backbone geometry, (e.g., in the case of the extended structure of a disordered chain), the neighboring side chains might point to the opposite direction while the second ones can be located in closer proximity to the lysine targeted for cross-linking and thus have to be considered as well. Supplementary Table S3 shows all the Lys residues with their neighbors and the frequency of their participation in the MS-identified cross-links. A frequency logo of five residue sequences with the lysines in center is shown in Figure 5A, revealing that there are some non-random motifs of yet unknown function around the lysines in the α-syn sequence. Calculating a frequency logo on the same pentapeptides of α-syn weighted with their occurrence as cross-linkable lysines in the MD simulation (by distance and accessibility, see Figure 4B), the logo shows an amino acid occurrence similar to the simple frequency in the sequence (Figure 5B) with some changes in the order but small changes in the actual ratios. It reveals that MD made no distinction between the lysines to be cross-linked. To the contrary, the frequency logo weighted by the ratios determined in the cross-linking reactions by MS revealed large changes with increased frequency of the KTKEG sequence (Figure 5C). There are 4 lysines with KXKXG sequence environment out of the 15 lysines (27%); however, they participate in 79% of the cross-links on one or both sides, and, if we include the N-terminal amino group, it adds up to 93%. In summary, there is a strong preference for cross-linking of the N-terminal and of the lysines in the center of KXKXG sequences. Oppositely, negatively charged residue is not preferred at the +2 position (Figure 5C).

### 2.5. Effect of the Introduced Cross-Links on the Fibrillation and Aggregate Morphology of α-Syn

The cross-linking agents chosen for our tests were DTSSP, BS3, BS(PEG)_5_, and EDC, as mentioned in the previous sections. Cross-linking reactions were optimized to form intramolecular bonds within α-syn. As the cross-linking reactions resulted in samples that were heterogenous in the cross-linked amine pairs, we tried to separate the various cross-linked α-syn species. Unfortunately, this proved to be a difficult procedure resulting in a low amount of purified samples hardly containing one type of cross-link. However, in comparison to the amyloid structures revealed, the MS-identified cross-links in the monomers are mostly incompatible with α-syn amyloids, especially the cross-links with the highest frequency, and thus the heterogeneous, non-separated cross-linked samples can be usable to test our hypothesis on the inhibitory effect of cross-linking.

**Figure 6:**
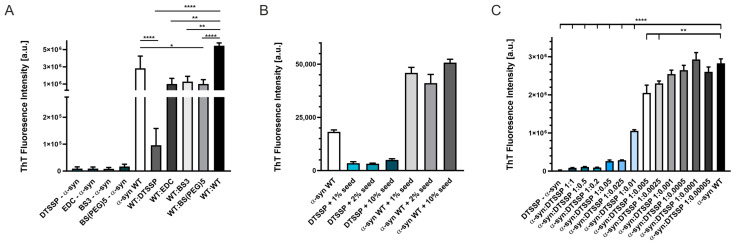
Aggregation studies of WT and cross-linked α-syn as followed by ThT fluorescence. (**A**) 100 μM α-syn samples cross-linked with DTSSP, EDC, BS3, and BS(PEG)^5^, in themselves, were not prone to form fibrils at 37 °C, 500 rpm, 48 h in PBS buffer, pH 7.4. ThT fluorescence intensities of WT (unmodified) and cross-linked α-syn mixtures (1:1 molar ratio, 100 μM each) are also shown, revealing significant inhibitory effects, with effectivity depending on the cross-linker. (**B**) Aggregation of DTSSP-cross-linked α-syn and WT α-syn as reference was further studied by the addition of preformed amyloid fibrils of WT α-syn as seeds at 1, 2, and 10% molar fraction. A 200 µM cross-linked α-syn showed low ThT fluorescence intensities even in the presence of seeds, while WT α-syn aggregated efficiently in the 48 h experiment. (**C**) DTSSP-cross-linked α-syn proved to be an effective inhibitor of fibrillation of WT α-syn as shown here by adding it to 200 µM WT α-syn at various molar ratios in the range of 1:1–1:0.0005. Statistical analyses are based on two-sample unpaired two-tailed *t*-tests. Based on the *t*-test, asterisks show: **** *p*-value <0.0001; ** *p*-value <0.005; * *p*-value <0.05. Note that the instrument used to measure ThT fluorescence in (**B**) was different from that of (**A**,**C**) resulting in different ThT fluorescence intensities.

First, cross-linked α-syn samples were tested for aggregation by ThT fluorescence assay. There was no aggregation observed in a two-day experiment at 100 μM of protein concentration for any of the cross-linkers (Figure 6A). We also tested the possible inhibitory effect of the cross-linked samples on the aggregation of WT α-syn and found significantly lower final ThT fluorescence intensities in the presence of cross-linked α-syn as compared to samples containing only WT α-syn. DTSSP-cross-linked α-syn showed the highest effect, almost fully blocking the fibrillation of WT α-syn in equimolar ratio (Figure 6A). We also checked the effect of seeding on the aggregation of DTSSP-cross-linked α-syn using preformed WT α-syn amyloid samples as seeds at 1, 2, and 10% fractions. After 48 h, non-seeded and seeded WT α-syn samples all reached high ThT fluorescence intensities, while DTSSP-cross-linked samples showed low ThT intensities, even when incubated in the presence of 10% WT α-syn seeds. This observation supports further our hypothesis that cross-links formed within the disordered α-syn monomer are incompatible with the amyloid structure. We analyzed further the inhibitory effect of DTSSP-cross-linked α-syn on the WT protein by applying a concentration series of the cross-linked protein. The effect was significant even at 1:0.005 WT α-syn: cross-linked α-syn molar ratio (Figure 6C).

**Figure 7:**
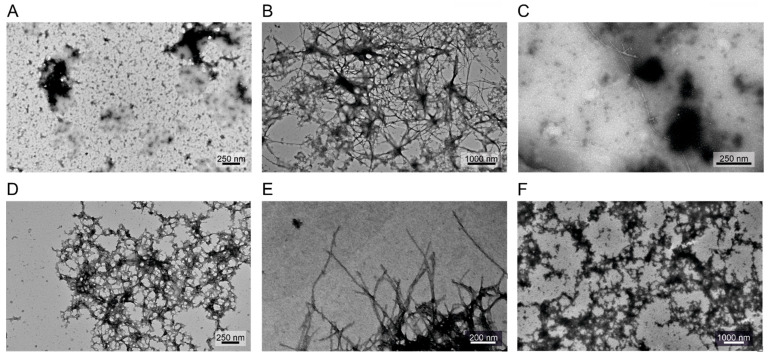
Transmission electron microscopy imaging of (**A**) DTSSP-cross-linked α-syn (100 μM), (**B**) WT (unmodified) α-syn (200 μM), (**C**) WT:DTSSP α-syn (100–100 μM, respectively), (**D**) WT:EDC α-syn, (**E**) WT:BS3 α-syn and (**F**) WT:BS(PEG)5 α-syn samples (100–100 μM, respectively). No fibril formation could be observed in samples containing DTSSP-cross-linked α-syn.

Supplementing the ThT assay results, the morphology of the possibly formed fibrils has been investigated by transmission electron microscopy (TEM) (Figure 7). Based on the obtained images, we concluded that the cross-linked samples have almost no observable fibril content, which is in good agreement with the ThT fluorescence results. However, in the presence of a 1:1 molar ratio of WT α-syn, the different cross-linked samples showed some abnormal collided formations in which uranyl acetate accumulates. These formations are not identical to the fibrils formed by WT α-syn itself and do not give a large fluorescent signal in ThT assay. Presumably WT α-syn molecules started to assemble but due to breaks caused by incorporation of the cross-linked molecules, the corresponding fibrils did not form.

## 3. Discussion

The aggregation and fibrillation of amyloidogenic proteins are associated with numerous degenerative diseases in the human body [10]. These proteins can be classified in different groups depending on the structural state of the native, monomeric protein. There are peptides which usually have hydrophobic properties and are produced by proteolytic cleavage in the body, such as amylin and the amyloid-β peptide. On the opposite end, there are globular, highly ordered proteins, which can be destabilized under certain environmental conditions, and their conformation undergoes a transition to a highly β-structured polymeric state of the amyloid fibrils (β_2_-microglobulin, transthyretin, Ig light chain) [10]. A special class of amyloidogenic proteins are natively disordered proteins, which are characterized by highly dynamic conformational ensembles. They have good solubility; however, they are still prone to form different types of aggregates and amyloid fibrils. α-Synuclein is an IDP which plays a role in synaptic vesicle trafficking in neurons [26]. However, upon aggregation and amyloid formation, the protein is deposited in the brain and is responsible for Parkinson’s disease and other synucleinopathies [21,22]. Due to its disease relation, blocking α-syn aggregation is receiving high attention. Because of the high conformational multiplicity, it is difficult to target the monomeric state with peptide or small molecule binders [27,28,29,30,31]. The amyloid formation is coupled with a large structural transformation to the highly non-native, cross-β-sheet structure.

There are few studies in the literature on α-syn using cross-linking. Selkoe and co-workers used cross-linking in vivo to study the oligomeric state of α-syn [32]. Lucas and co-workers investigated the metal ion-induced structural alterations in α-syn by photo-chemical cross-linking and pointed out the different coordination and the related molecular mechanisms for copper and iron [33]. Schmid et al. determined the effect of tissue transglutaminase catalyzed cross-linking on the oligomerization, fibrillization, and membrane binding of α-syn. Cross-linking at Gln79 and Gln109 had inhibitory effects on α-syn fibrillogenesis [34].

Cross-linking of α-syn might occur naturally in vivo. Depending on the cross-linked structural form of the molecule (disordered vs. aggregated species), amyloid formation can be inhibited or the fibril structure can be promoted and stabilized. Nemes et al. studied the effect of the transglutaminase-mediated intramolecular cross-linking of α-syn, which was identified between Gln99 and Lys58 in Lewy bodies. In vitro cross-linking by transglutaminases produced a heterogeneously cross-linked solution, which inhibited amyloid formation [35]. However, in the presence of phosphatidylserine-rich membranes and calcium, the formation of the Gln99–Lys58 cross-link was preferred, which triggered amyloid formation. In contrast, the cross-link between residues 99 and 10 had an inhibitory effect. In their recent work, Sahin et al. modeled the effect of oxidative stress on the α-syn structure and self-assembly. They found that mild oxidation led to intramolecular cross-linkages, compaction of the monomer molecule, and the inhibition of amyloid formation by steric hindrance, suggesting that mild oxidation has a role in preventing amyloid formation [36].

In the present study, we introduced structural constraints to the monomeric α-syn molecule by cross-linking and studied its effect on the aggregation of the protein and investigated the possible inhibitory effect of cross-linked α-syn molecules on the native α-syn. First, we carried out an in silico structural analysis on native monomeric α-syn and on the available high-resolution amyloid structures to predict the potential cross-linking sites and their usability to block the aggregation of the protein. How to study the structure of the IDP α-syn monomer was an intriguing task. First, we tried to use the eight representative native α-syn structures published by Chen et al. [24] and surveyed them for amines that lie within predefined cross-linking distances from each other. The observed amine pairs were compared to the potential cross-linking pairs in the structures of α-syn amyloid fibrils downloaded from the PDB (Supplementary Table S1). The results revealed that most of the potential cross-links in the native monomeric structures are non-compatible with the amyloid forms; therefore, they might be suitable as constraints to block amyloid formation (Figure 1, Supplementary Figure S1). In the second experimental step of the study, α-syn was cross-linked with several cross-linkers (Supplementary Figure S2) under conditions where the reactions occur intramolecularly (Supplementary Figure S3) and mostly one cross-link is formed per molecule. The cross-linked samples were analyzed by LC–ESI–MS and LC–ESI–MS/MS techniques for native and bottom-up analyses, respectively (Figure 2, Supplementary Table S2 and Supplementary Figures S5–S7) and proved to be heterogeneous in the cross-links (Supplementary Figure S4). Taking advantage of the extra ion mobility separation opportunity with the Cyclic IMS instrument, it was possible to increase cross-link identifications of the heterogeneous chemically modified α-syn molecules. In previous studies, Cyclic IMS has been described to enhance overall protein coverage and peptide identifications in proteomics studies [37]. Furthermore, the NHS ester cross-linkers we used tend to dissociate upon CID fragmentation at their cross-linker–lysine amide bond resulting in diagnostic, cross-linker-specific ions, which support the unambiguous identifications [9]. Additionally, the DTSSP cross-linker is prone to dissociate at the linker’s S–S bond as well [38] which further complements CSM detections. Fragmentation of the linker is potentiated when additional IMS was applied before CID fragmentation; thus, higher scoring and on average enhanced cross-link recognition can be obtained. For the above reasons, DTSSP was proved to be an efficient cross-linker, and it was possible to identify 60 different cross-linked amine pairs that were observed with different frequencies (Supplementary Table S2). The experimental results overlapped only in half with the expected cross-linkable amine pairs from the eight structures (Figure 1), revealing that those representative structures with a simple amine-distance analysis are insufficient to depict the entire conformational ensembles of α-syn. To provide a better description of the dynamic conformational ensembles, we carried out MD simulation with α-syn and analyzed the entire trajectory for potential amine cross-link distances and determined their frequencies (occurrence in % of time) (Figure 4). All the possible 120 amine pairs showed distances within the cut-off for cross-linking at least for a few frames in the trajectory, which assume a significantly larger conformational space than that characterized by the eight structures. However, the calculated occurrences showed no correlation with the experimentally observed cross-linking frequencies (Figure 4). We tried to carry out a more sophisticated analysis on the trajectory by examining if there is a steric hindrance between the two amines (see Section 4). We found an altered frequency for the potential cross-links; however, there was still no real correlation with the experimental frequencies. These results reveal that the in silico analysis is unable to reliably describe the intrinsically disordered α-syn chain and to predict its complex behavior. Borchers and co-authors studied the structure of native α-syn using the ABAS cross-linker and identified 10 cross-links using MS techniques [39]. These cross-links were incompatible with the α-syn NMR structure of Ulmer et al. [40], and they concluded that the in-solution structure of α-syn may be significantly different from that of the micelle-bound structure of monomeric α-syn.

Analyzing further the experimental results, we found that the sequential environment (neighboring residues) has a large effect on the cross-linking frequencies. Not surprisingly, the amino terminal with the lowest *pK*_a_ value participated at the highest frequency in the crosslinks. Among the Lys side chains, the one in the middle of the KXKXG motif showed the highest frequency, whereas the negative charge was not preferable at the end of the motif (Figure 5, Supplementary Tables S2 and S3). These results revealed that cross-linking efficiency in the natively disordered chain of α-syn is rather determined by the physico-chemical properties/local environment, affecting the chemical reaction rate and is difficult to predict simply based on distances in the structure.

Compared to the amyloid structures (Supplementary Table S1), the experimentally identified cross-links and their frequencies in the samples assumed that although the samples are heterogeneous, most of the cross-links are incompatible with the amyloid structures and can block the fibrillation of the protein. Indeed, none of the cross-linked samples showed aggregation in the ThT fluorescence assays (Figure 6).

An important observation is that the DTSSP-cross-linked α-syn samples displayed a significant inhibitory potency at sub-stoichiometric concentrations on the aggregation of the unmodified α-synuclein even at a 0.01:1 cross-linked/native ratio (Figure 6C). TEM images also proved the inhibitory effect of DTSSP-cross-linked α-syn (Figure 7). In order to inhibit aggregation at sub-stoichiometric ratios, the cross-linked molecule has to bind with high affinity to α-syn aggregates and block the further growth by not being able to transform into the amyloid conformation, which is assured by the cross-linking.

Here, we used cross-linked samples containing a heterogeneous mixture of intramolecularly cross-linked molecules (see Supplementary Table S2 for components and their frequencies). In the future, by selective purification, cross-links with the most beneficial effects could be sorted out.

In this work, we studied the effect of cross-linking on aggregation and fibrillation on α-syn as model protein. It is a question whether our results have therapeutic or in vivo relevance. The inhibitory effect at sub-stoichiometric concentrations is promising in this regard, and moreover, the cross-linked α-syn is probably non-immunogenic. However, we would rather think that such cross-linked α-syn, instead of a highly dynamic native molecule, might be a good target for screening of small molecules, peptides, or proteins that will bind and stabilize the “non-amyloid” conformation of α-syn. Moreover, such a conformation might have an inhibitory effect on the aggregation of α-syn.

## 4. Materials and Methods

### 4.1. Expression and Purification of α-Synuclein

*E. coli* BL21 Star (DE3) pLysS cells were transformed with pT7-7 plasmid containing the WT α-synuclein (*H. sapiens*) cDNA sequence, grown in LB medium containing 100 µM/mL ampicillin and induced with 1 mM IPTG at OD600 = 0.6 for 3 h at 37 °C. Following cell harvesting by centrifugation at 7000× *g* for 20 min at 4 °C, monomeric α-syn was purified as described earlier [41,42]. After lyophilization, the protein was kept at −20 °C until usage.

### 4.2. Cross-Linking Reactions of α-Synuclein by Chosen Agents and Optimization

Reagents were products of Sigma-Aldrich (Merck Life Science Kft., Budapest, Hungary or Merck KGaA, Darmstadt, Germany). First attempts were carried out by general protocols from the manufacturers for DTSSP (Sigma-Aldrich, 803200 (3,3′-dithiobis(sulfosuccinimidyl propionate))), BS3 (Sigma-Aldrich, S5799 Suberic acid bis(3-sulfo-*N*-hydroxysuccinimide ester) sodium salt), EDC (Sigma-Aldrich, 8.00907 *N*-(3-Dimethylaminopropyl)-*N*′-ethylcarbodiimide hydrochloride) and Sulfo-NHS (Sigma-Aldrich, 56485 *N*-Hydroxysulfosuccinimide sodium salt), BS(PEG)5 (Sigma-Aldrich, 803537 PEGylated bis(sulfosuccinimidyl)suberate). Reactions were optimized for intramolecular cross-linking, using an adapted protocol from Pál-Gábor et al. [43]. Samples were incubated with cross-linkers at a protein to cross-linker ratio of 2:1 (*w*/*w*) for 1 h at 37 °C. The reactions were quenched by addition of 100 mM TRIS, pH 7.4, at a 50-fold molar excess to the cross-linker. The quenching reaction was allowed to proceed for 30 min. After quenching, free cross-linkers were removed on a PD-10 column (Sephadex G-25M, GE17-0851-01). Cross-linked α-syn solutions were concentrated and transferred into MQ by centrifugal filtration (Amicon Ultra–3K, Merck Millipore Ltd., Carrigtwohill, Ireland). Protein concentration was measured at 214 nm [44] and 205 nm [45]. The monomeric state of the proteins was verified using SDS PAGE under non-reducing conditions.

### 4.3. Protein Measurement with LC–MS and Data Analysis

For all MS analyses chemicals were from Sigma-Aldrich (Munich, Germany) except where indicated otherwise.

Approximately 2 nmol of cross-linked α-syn dissolved in MQ water was injected into a Waters Acquity I-Class UPLC system connected to a Waters Select Series Cyclic IMS (Waters Corporation, Milford, UK), hybrid quadrupole–TOF mass spectrometer. Liquid chromatographic separation of the protein and its modified variants was performed on an Acquity UPLC BEH300 C4 column, 300 Å, 1.7 μm, 1 mm × 150 mm. Mobile phase (A) was composed of 0.1% trifluoroacetic acid (TFA) in water; mobile phase (B) was composed of 0.1% TFA in acetonitrile. The elution method at a flow rate 400 µL/min included the following gradient: 1 min: 5% B, 12 min: 60% B, 12.5 min: 90% B at 80 oC. MS data acquisition was performed with the following parameters: *m*/*z* 350–2000, V-mode, scan time: 0.3 s, single Lock Mass: leucine–enkephalin.

Data were analyzed using Waters MassLynx 4.2 and spectra were inspected manually.

### 4.4. Sample Preparation for LC–MS/MS Analysis, Measurement, and Data Evaluation

Protein samples of 20–20 µg were denatured with 0.01% Rapigest (Waters Corporation, Milford, UK). Then (except for the DTSSP-cross-linked samples), they were reduced with 10 mM of TCEP at 37 °C for 30 min and alkylated with 55 mM of iodoacetamide at room temperature for 30 min in the dark. Samples were treated with trypsin (Promega Corporation, Madison, WI, USA) at an enzyme-to-protein ratio of 1:100 (*wt*/*wt*). Digestion was performed at 37 °C for 4 h. Enzymatic digestion was quenched by adding formic acid to reach a final concentration of ~1%. Peptides were purified and enriched using Pierce C18 Spin Columns according to the manufacturer’s description. Eluted samples were dried using SpeedVac and kept at −20 °C until LC–MS/MS analysis.

Samples were reconstituted in a 25 µL 2% ACN, 0.1% formic acid solution before injection into a Waters Acquity I-Class UPLC system connected to a Waters Select Series Cyclic IMS (Waters Corporation, Milford, UK), hybrid quadrupole–TOF mass spectrometer with high resolution and high mass accuracy, equipped with a cyclic ion mobility separation device. For peptide separation, samples were loaded on Waters Acquity Peptide CSH column (1.7 µm, 1 mm × 150 mm) or multi-step gradient elution. Mobile phase (A) was composed of 0.1% formic acid in water; mobile phase (B) was composed of 0.1% formic acid in acetonitrile. The elution method at a flow rate 20 µL/min included the following: 1 min: 5% B, 45 min: 35% B, 46 min: 85% B at 45 °C. MS data acquisition was performed with the following parameters: *m*/*z* 50–2000, V-mode, scan time: 0.5 s, single Lock Mass: leucine–enkephalin. HDMS^E^ fragmentation was performed in the transfer cell: low energy: 6 V, high energy: ramping 19–45 V, after one ion mobility separation cycle. MS^E^ fragmentation was performed in the transfer cell: low energy: 6 V, high energy: ramping 19–45 V.

Raw data were converted to mzML file format and searched for contaminants using Waters ProteinLynx Global Server (PLGS) v3.0.3 software in the SwissProt database. Parent and fragment ion tolerance was set to 20 ppm and 30 ppm, respectively. Digestion enzyme was trypsin and missed cleavages were set to 2. Carbamidomethyl modification on cysteines was set as fixed modification and for variable modifications oxidation on M. The false discovery rate was 2%, and only proteins with a minimal probability of 95% were counted. Processing parameters were the following: low energy threshold: 200 counts; elevated energy threshold: 20 counts; minimum fragment ion matches per peptide: 3; minimum fragment ion matches per protein: 7; minimum peptide matches per protein: 2. The cross-link analysis was performed using Protein Prospector. The digestion enzyme was set to trypsin with 2 missed cleavages; constant modification was carbamidomethyl (C) and variable modifications were: acetyl (Protein N-term), Gln to pyro-Glu (N-term Q), Met-loss (Protein N-term M), Met-loss + Acetyl (Protein N-term M), oxidation (M). Cross-linkers were set to EDC, DSP for DTSSP, DSS (disuccinimidyl suberate) for BS3, and BS(PEG)5 was set as user-defined cross-link, bridge components: C14O7H22, linking at lysine residues and protein N-terminus. The parent ion tolerance was set to 20 ppm; the fragment ion tolerance was 30 ppm; and the instrument was set to ESI-Q-TOF. The database was created with proteins previously found in the SwissProt search, random concatenated; the FDR cut off was 1%. The mass spectrometry-based proteomics data have been deposited in the ProteomeXchange Consortium via the PRIDE [46] partner repository with the dataset identifier PXD042501.

### 4.5. MD Simulations and Trajectory Analysis for Potential Cross-Linking Pairs

The monomer structure of α-syn was subjected to MD simulations as implemented in GROMACS [47], using the AMBER-ff99SB*-ILDNP force field [48]. As initial monomeric conformation, we used the third model of the ensemble structure of the α-syn monomer determined using FRET data and discrete MD simulations [24] (PDBDEV_00000082, Model #3). The system was solvated by ~10,032 water molecules with TIP4P parametrized water molecules [49]. The total charge of the system was neutralized, and the physiological salt concentration was set by placing Na^+^ and Cl^−^ ions. Energy minimization of starting structures was followed by sequential relaxation of constraints on protein atoms in three steps and an additional NVT step (all of 200 ps) to stabilize pressure. One µs trajectory of NPT simulations was carried out at 283 K and at 1 bar by collecting snapshots at every 20 ps.

The possibility of DTSSP cross-linking was investigated in silico based on the distance between the N-terminal and lysine side chains. The distance was calculated using the amino N atom of the N-terminal and of the lysine side chains, using in-house Matlab scripts. To calculate the distances in the monomeric form, first we used the 8 structural models reported by Chen et al. [24], and for the amyloid form, we used structures from the PDB (Supplementary Table S1). For intramolecular cross-linking with the DTSSP cross-linker, we used a cut-off of 15 Å in all cases. These distances were also calculated for the MD simulations, and we determined for each N–N pair the frequency (percentage) in the entire 1 µs trajectory (5001 frames), when the pair is within the cut-off distance.

We have further sorted the amine pairs that lie within cut-off distance for steric availability for cross-linking, i.e., whether there are molecule parts between them hindering a possible reaction. To check this in a simple and rapid way with low computational power needs, we placed three spheres with a radius of 2.5 Å on the line connecting the two nitrogen atoms (Supplementary Figure S8). These spheres may overlap if the space between the two N atoms is less than 15 Å, and we use only one sphere if the distance between the two nitrogen atoms is less than 5 Å. The other atoms of the molecule were represented by spheres of their van der Waals radii. If such a sphere has intersection with one of the spheres between the two N atoms, the case was treated as blocked for cross-linking. Note that the atoms in the space “behind” the nitrogen atoms were not treated as blockers (Supplementary Figure S8).

### 4.6. α-Synuclein Aggregation and Thioflavin T Fluorescence Assay

Purified α-syn was dissolved in PBS or 20 mM Na-phosphate, 100 mM NaCl (pH 7.4), at a concentration of 400 µM. Cross-linked α-syn samples in MQ water were adjusted to the required buffer conditions. After monomeric WT and cross-linked α-syn solutions were diluted to the required working concentrations (100–200 µM) in the used ratios, the samples were split into at least three replicates in Eppendorf tubes containing each a Teflon polyball (1/800 diameter) and placed into a shaker (Eppendorf ThermoMixer C, Eppendorf A.G., Hamburg, Germany). Aggregation processes were carried out at 37 °C, 500 rpm for 48–100 h. For the thioflavin T (ThT) fluorescence assay, reaction mixtures were mixed with 20 μM ThT. The fluorescence intensity was measured on a SPEX FluoroMax fluorometer (SPEX Industries, Edison, NJ, USA) with excitation and emission wavelengths of 445 and 485 nm, respectively, at 25 °C.

For seeded aggregation measurements, a final concentration of 200 μM DTSSP-cross-linked and WT α-syn samples were used in 20 mM NaH_2_PO_4_, 100 mM NaCl (pH 7.4) containing 20 µM ThT and 0.05% sodium azide. Seeds were prepared with 3 min ultrasonication using a Branson Ultrasonic B200 Equipment of a 200 μM WT α-syn amyloid solution (grown in a shaking incubator for 2 days at 500 rpm, 37 °C), then added to the freshly prepared monomer solutions at 1–10% final concentration in a 96-well plate (polystyrene non-binding microplate, Greiner, sealed). The ThT fluorescence intensities were measured using a plate reader (Synergy H4 Hybrid Reader, BioTek Instruments, Winooski, VT, USA). Instrument settings: xenon lamp, 37 °C, 48 h, measurements every 30 min, rapid shaking constantly, excitation 445 nm, emission 485 nm, gain 80.

For dose-response measurements, the aggregation of 200 μM α-syn WT monomer solutions were monitored in the presence of different DTSSP-cross-linked α-syn fractions (between 1:1–1:0.00005, equal to 200 μM–10 nM cross-linked protein concentrations). Samples were aggregated in a shaker incubator (Eppendorf ThermoMixer C, Eppendorf A.G., Hamburg, Germany) at 500 rpm at 37 °C for 48 h. Fluorescence intensities of 20 µM ThT were measured on a FluoroMax fluorometer (SPEX Industries, Edison, NJ, USA) with excitation and emission wavelengths of 445 and 485 nm, respectively, at 25 °C.

### 4.7. Transmission Electron Microscopy (TEM)

Aggregated WT, cross-linked, and mixed α-syn samples from final point reactions were collected and diluted to a protein concentration of 10–12 µM. Samples of 5 µL were placed on carbon-coated formvar copper grids and incubated for 2 min. Then, samples were carefully removed using the edge of a filter paper, and 5 μL 1% (*w*/*v*) of uranyl acetate was applied for negative staining. After 1 min, the excess staining solution was removed, and the grids were air-dried. The samples were imaged using a Jeol J1100 transmission electron microscope (JEOL, Tokyo, Japan) operating at an accelerating voltage of 80 kV. At least 30 fields were screened from parallel samples to obtain representative images.

## Data Availability

The data in this study are readily available upon reasonable request to the corresponding author.

## Supplementary Materials

The supporting information can be downloaded at https://www.mdpi.com/article/10.3390/ijms241713403/s1.
