# Supplementary material for: Cross-Linked α-Synuclein as Inhibitor of Amyloid Formation
Source: Int J Mol Sci. 2023 Aug 29;24(17):13403. doi: 10.3390/ijms241713403 (PMC10487470; doi:10.3390/ijms241713403)
Supplement: Supplementary file 1 [file ijms-24-13403-s001.zip › ijms-2527379-supplementary.pdf]

Supporting Information

Nikoletta Murvai, Gabriella Gellen, András Micsonai, Gitta Schlosser, and József Kardos*

*Correspondence: kardos@elte.hu

Supplementary Table S1. PDB IDs of amyloid structures of α-synuclein

PDB IDs					
2N0A	6OSM	7E0F	7V47	7XO3	8H03
6A6B	6PEO	7L7H	7V48	8A4L	8H04
6CU7	6PES	7LC9	7V49	8A9L	8H05
6CU8	6RT0	7NCA	7V4A	8ADS	8HO0
6FLT	6RTB	7NCG	7V4B	8ADU	
6L1U	6UFR	7NCJ	7WMM	8AEX	
6L4S	6XYO	7NCK	7WNZ	8BQV	
6LRQ	6XYP	7OZG	7WO0	8BQW	
6OSJ	6XYQ	7OZH	7XO1	8CE7	
6OSL	7C1D	7UAK	7XO2	8CEB	

Supplementary Table S2. Frequencies of the DTSSP cross-links identified by MS

Res. #1[a]	Res. #2[a]	Occ.[b]	Res. #1[a]	Res. #2[a]	Occ.[b]	Res. #1[a]	Res. #2[a]	Occ.[b]
0	6	7	6	96	1	23	58	1
0	10	5	6	97	1	23	60	2
0	12	15	10	12	2	23	80	2
0	21	5	12	21	2	23	97	3
0	23	7	12	23	8	32	34	1
0	32	8	12	32	2	32	43	2
0	34	6	12	34	7	32	45	1
0	43	7	12	43	2	32	97	1
0	45	8	12	45	3	34	45	9
0	58	4	12	58	2	34	58	3
0	60	5	12	60	3	34	60	7
0	96	2	12	80	3	34	96	1
0	97	4	12	96	2	34	97	2
6	12	3	12	97	2	45	58	3
6	21	2	21	34	1	45	60	3
6	23	2	21	97	1	45	80	2
6	32	1	23	32	1	45	97	3
6	34	1	23	34	9	58	97	1
6	45	1	23	43	2	60	80	2
6	60	1	23	45	4	60	97	1

[a] Cross-linked residue pairs and [b]their occurrences detected by MS. Residue 0 is for N-terminal amino group.

Supplementary Table S3. Occurrences of amines in cross-links determined experimentally or predicted by MD[a]

Position of amine	Sequence	Cross-link frequency	Occurrence in MD (%)
0	N-term.	83	4.33
6	FM**K**GL	20	7.71
10	LS**K**AK	7	5.22
12	KA**K**EG	56	11.34
21	AE**K**TK	11	3.56
23	KT**K**QG	41	12.02
32	AG**K**TK	17	15.25
34	KT**K**EG	47	5.49
43	GS**K**TK	13	11.61
45	KT**K**EG	37	4.38
58	AE**K**TK	14	8.95
60	KT**K**EQ	24	8.23
80	AQ**K**TV	9	7.47
96	FV**K**KD	6	4.72
97	VK**K**DQ	19	3.81
102	LG**K**NE	0	5.69

[a]Sequential neighbors of lysines (highlighted with bold "K" in the central position) and the frequency of lysines and the N-terminal amino group identified in cross-links by MS, and the time fraction (%) of the MD simulation a lysine and the N-terminal is within cross-linking distance of another amine with no steric hindrance for cross-linking.

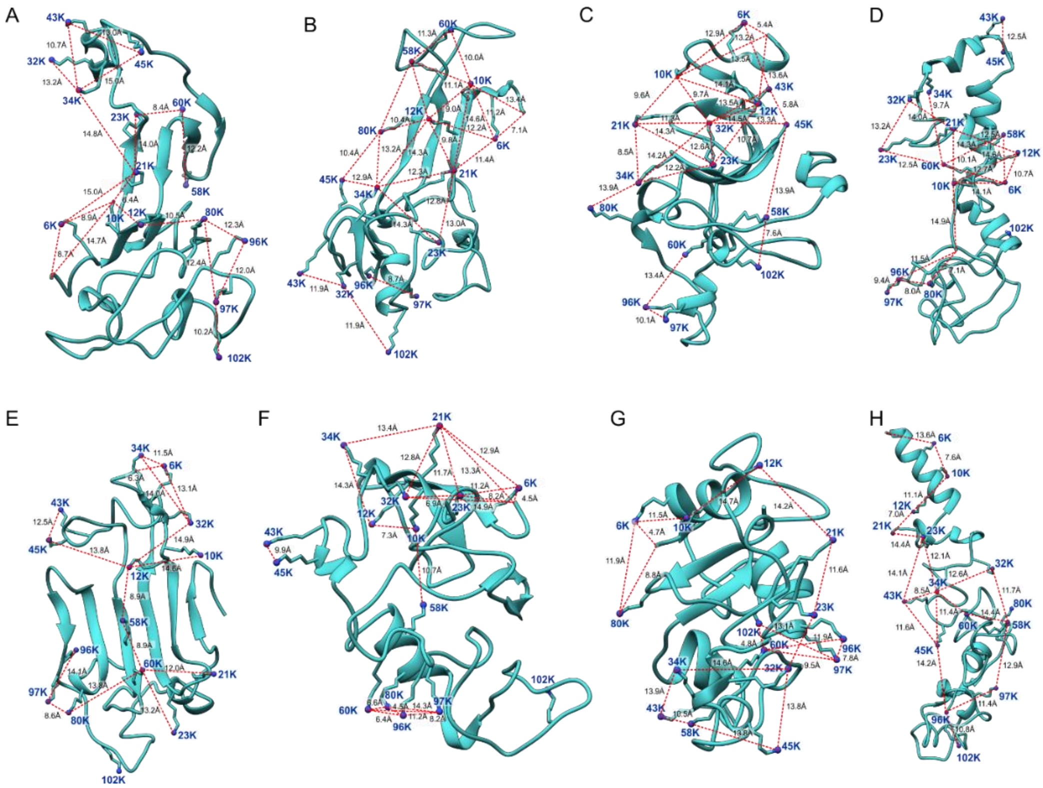

Figure S1. Amine distances in representative model structures of monomer α-syn. Dashed lines in red show amine pairs within 15 Å distance potentially suitable for cross linking. These pairs rarely can be found in amyloid structures of α-syn (see Section 2.1). Altogether, 60 pairs were identified, 33 of which were observed experimentally by cross-linking with DTSSP (see Section 2.4).

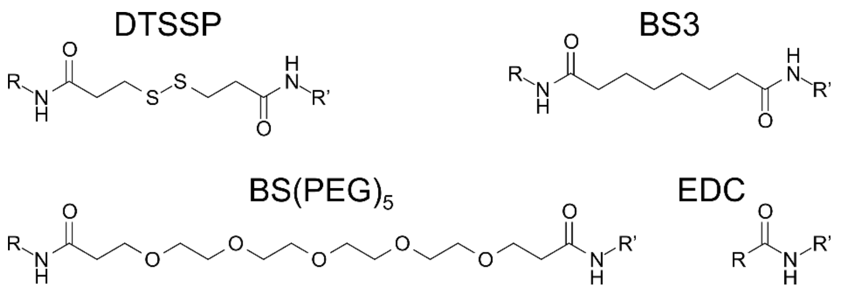

Figure S2. Cross-links applied in this study on α-syn. DTSSP, BS3, and BS(PEG)5 connect amines, while EDC links Lys to Asp or Glu. We carried out detailed studies using DTSSP.

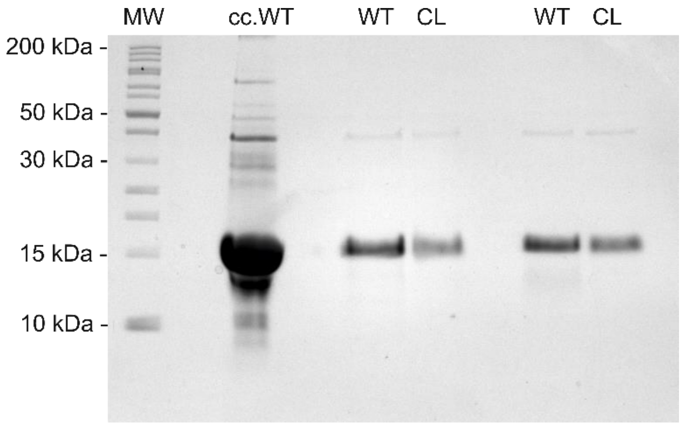

Figure S3. Cross-linking reaction on α-syn examined by non-reducing SDS-page. Cc. WT indicates concentrated unmodified α-syn sample containing high amount, ~10 μg protein. WT indicates samples without cross-linking, CL indicates samples cross-linked by DTSSP. (Left) reaction was carried out in MES buffer, pH 7.0, (right) reaction was carried out in PBS, pH 7.4. These results show that cross-links were formed only intramolecularly.

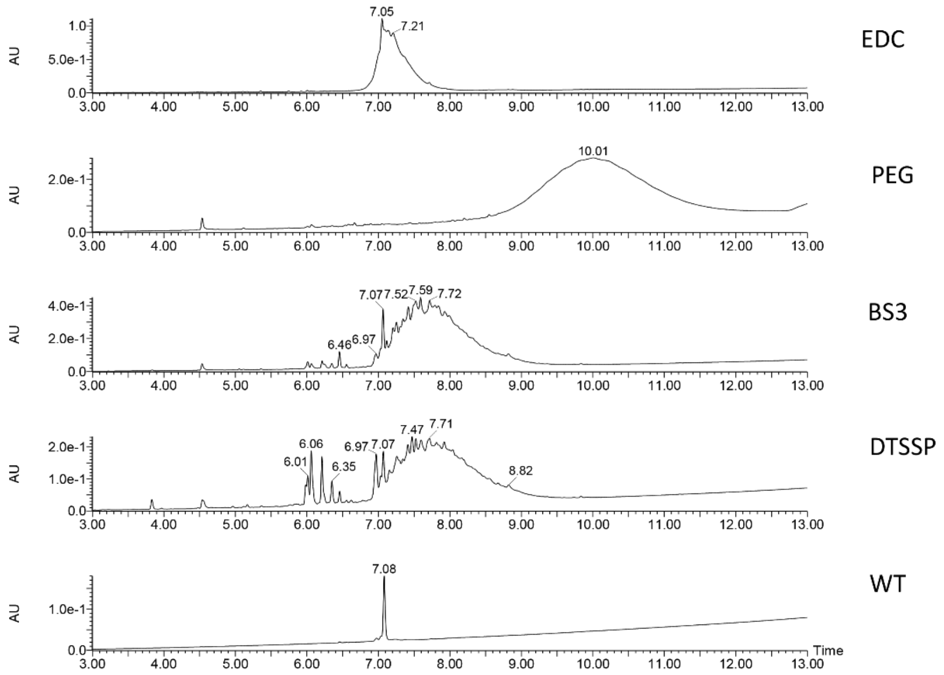

Figure S4. HPLC-UV chromatograms of cross-linked and unmodified α-syn. Comparison to unmodified α-syn (labeled as 'WT') shows a low fraction of the unmodified molecules, and the broad bands of cross-linked samples suggest the large heterogeneity in cross-linking. Liquid chromatography separation of the protein and its modified variants was performed on a C4 column using a 5-60% acetonitrile gradient (see *Material and Methods*).

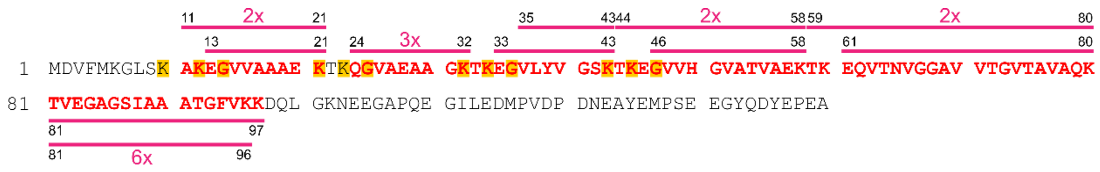

Figure S5. α-Syn fragments identified after trypsin digestion by LC-MS/MS technique. We have found a smooth coverage of the α-syn sequence, except for the 81-96 region. The four KXKXG sequence motifs are highlighted with yellow. The amino group of the central Lys of these, together with the N-terminal amino group (5 sites out of the possible 15) appeared in 93% of the detected cross-links at one or both sides, which, based on the above results, cannot be artifacts from the MS technique.

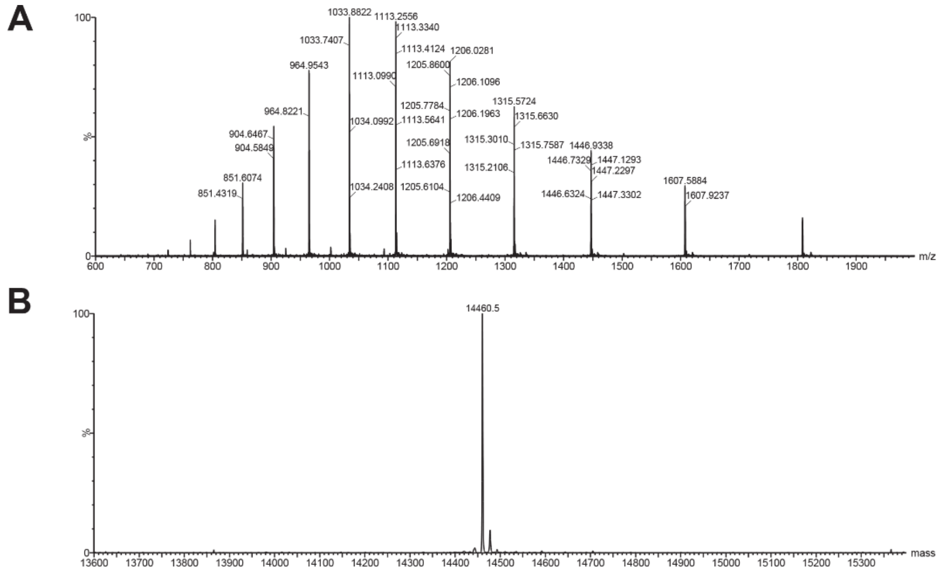

Figure S6. ESI-MS spectrum of the intact WT α-syn. (A) Full MS spectrum and (B) deconvoluted spectrum of WT α-syn showing the correct molecular weight.

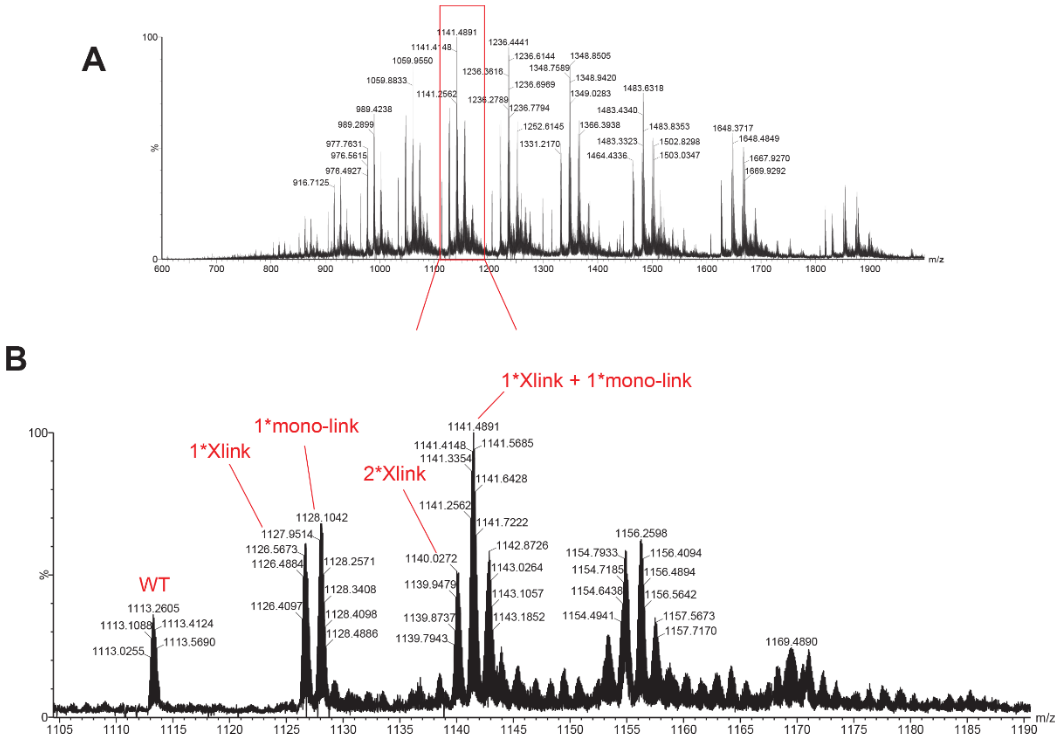

Figure S7. Full ESI-MS spectrum of the intact DTSSP cross-linked α-syn. (A) Full MS spectrum and (B) its enlargement showing the different species of modified α-syn. Large portion of the molecules have single cross-linking with or without an extra mono-link. At a small fraction, unmodified α-syn and molecules with multiple cross-links are also observable.

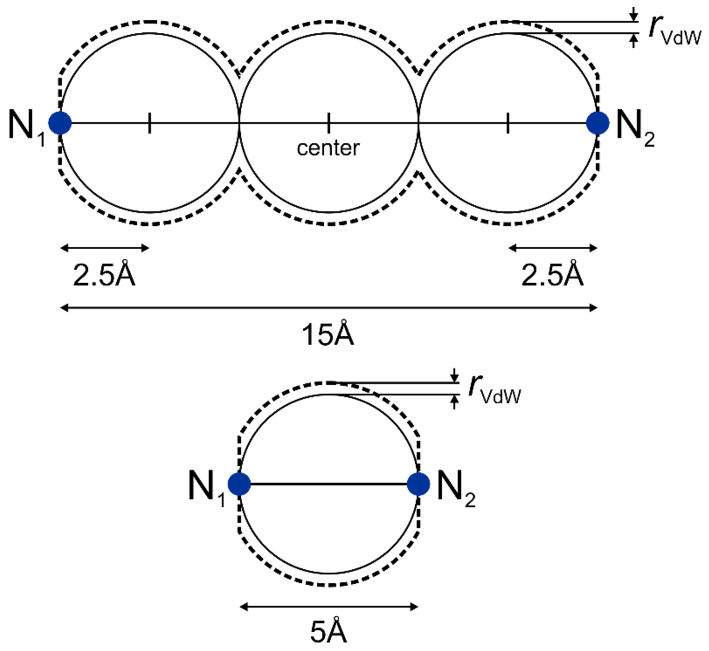

Figure S8. Examination if there is no steric hindrance for cross-linking. For a sterically realizable cross-link, the "non-occupied" space between the amines was verified as devoid volume of three overlapping spheres of 5 Å diameter positioned between the two nitrogen atoms as shown above and described in the *Materials and Methods* section. When the distance between the nitrogens is as low as 5 Å, the three spheres fuse to a single one. Using these spheres makes the calculation of steric hindrance in the structures of the MD trajectory reasonably fast.
